# A new stunt nematode, *Geocenamus chengi* n. sp. (Nematoda: Merliniinae) in the rhizosphere of tea (*Camellia sinensis*) from Zhejiang Province, China

**DOI:** 10.21307/jofnem-2020-042

**Published:** 2020-04-16

**Authors:** Munawar Maria, Wentao Miao, Pablo Castillo, Jingwu Zheng

**Affiliations:** 1 Laboratory of Plant Nematology, Institute of Biotechnology, College of Agriculture & Biotechnology, Zhejiang University, Hangzhou 310058, Zhejiang, P.R. China; 2 Institute for Sustainable Agriculture (IAS), Spanish National Research Council (CSIC), Campus de Excelencia Internacional Agroalimentario, ceiA3, Avenida Menéndez Pidal s/n, 14004 Córdoba, Spain; 3 Ministry of Agriculture Key Lab of Molecular Biology of Crop Pathogens and Insects, Hangzhou 310058, P. R. China

**Keywords:** DNA sequencing, *Camelliae sinensis*, morphology, morphometrics, nematode, new record, species, phylogeny, scanning electron microscopy

## Abstract

The tea plant is native to China; the country has the greatest tea production areas in the world. In an attempt to investigate the nematode biodiversity associated with the tea plantations of Hangzhou, Zhejiang Province, a population of stunt nematode was detected. This group of nematodes is comprised of migratory ecto-parasites of roots and can subsist on a variety of host plants. Therefore, the detected population was studied carefully using the integrative taxonomy approach and identified as a new species of genus *Geocenamus*. *Geocenamus chengi* n. sp. can be characterized by females having six incisures in the lateral field; labial region is dome shaped and slightly offset from the rest of the body having four to five annuli; head framework is weakly developed; deirids are absent; excretory pore is located at the anterior region of basal pharyngeal bulb. Under SEM, the vulva is a transverse slit, vulval lips are elongated and ellipsoidal with epiptygma. The tail is annulated, elongated, and conical having bluntly pointed tip and a terminal hyaline region that forms 21 to 33% of the tail length. Spicule is 22 to 25 μm long, gubernaculum is saucer shaped; bursa is crenated covering the tail until the hyaline tail region. Morphologically, the species is close to *G. circellus*, *G. joctus*, *G. loofi*, *G. ordinarius*, *G. processus*, *G. tetyllus*, and *G. tortilis*. Phylogenetic relationships of the new species based on D2-D3 expansion domains of 28 S, ITS, and 18 S rRNA genes indicated that *G. chengi* n. sp. clustered in a separate clade with *G. vietnamensis*.

The tea plant is native to China; this country has the highest tea production and consumption rate in the world. More than 100,000 hectares of tea acreage are located in seven Chinese provinces: Yunnan, Sichuan, Fujian, Hubei, Zhejiang, Guizhou, and Anhui (Yao and Chen, 2012). In an attempt to investigate the nematode biodiversity associated with the tea plantations of Hangzhou, Zhejiang Province, a population of stunt nematode of subfamily Merliniinae was detected. This family comprises migratory ectoparasites of roots; merlinid nematodes are root feeders and can subsist on a variety of host plants (Siddiqi, 2000). The detected population of stunt nematode was examined carefully, and the morphological characterization indicated that this population belongs to the genus *Geocenamus* (Thorne and Malek, 1968).

**Figure 1:**
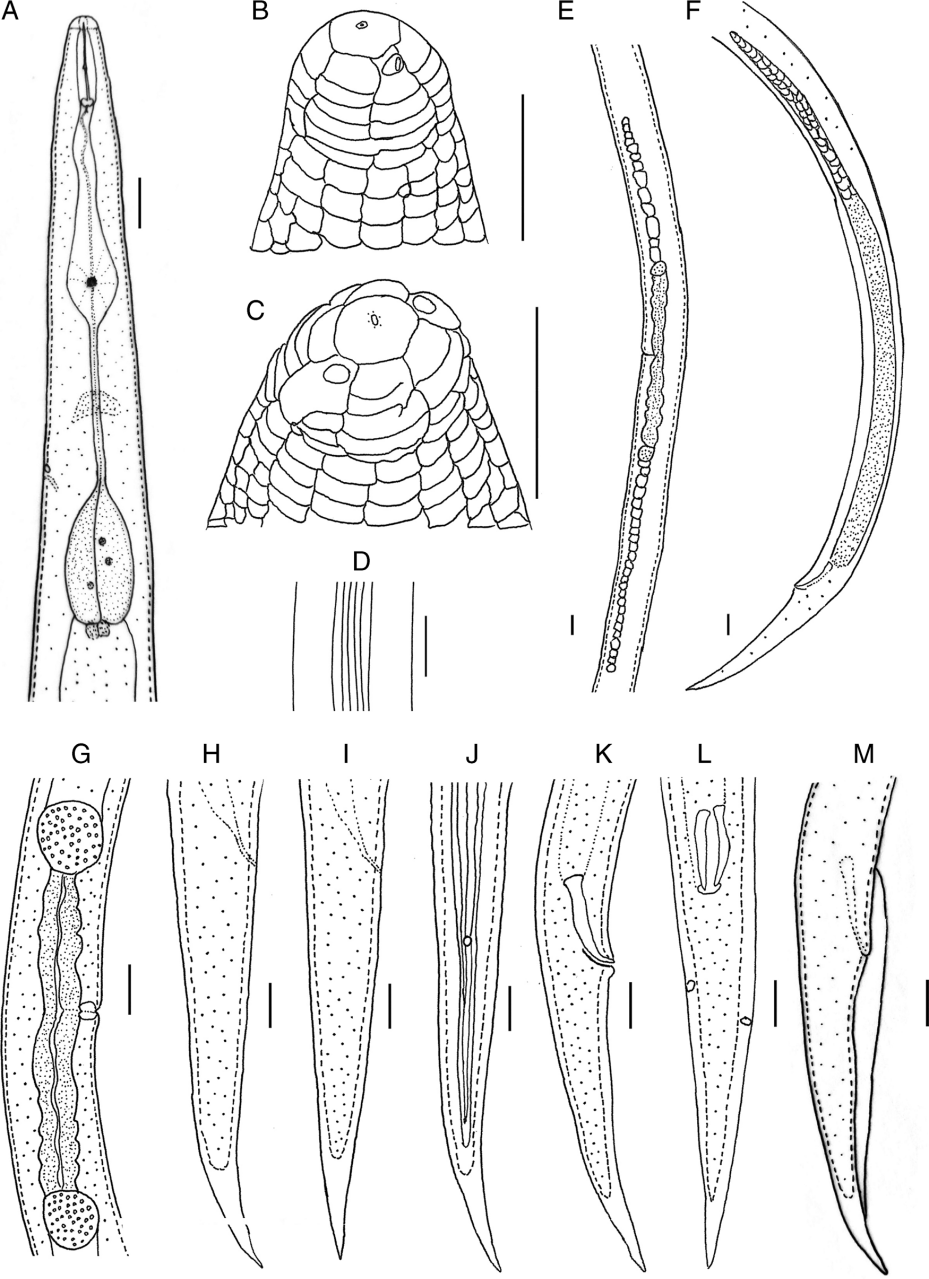
Line drawings of *Geocenamus chengi* n. sp. A: pharyngeal region; B, C: en face view; D: lateral lines: E: female gonad; F: male gonad; G: vulval region; H-J: female tails: K-M: male tails (Scale bars = A; D-M = 10 μm, B,C = 5 μm).

**Figure 2:**
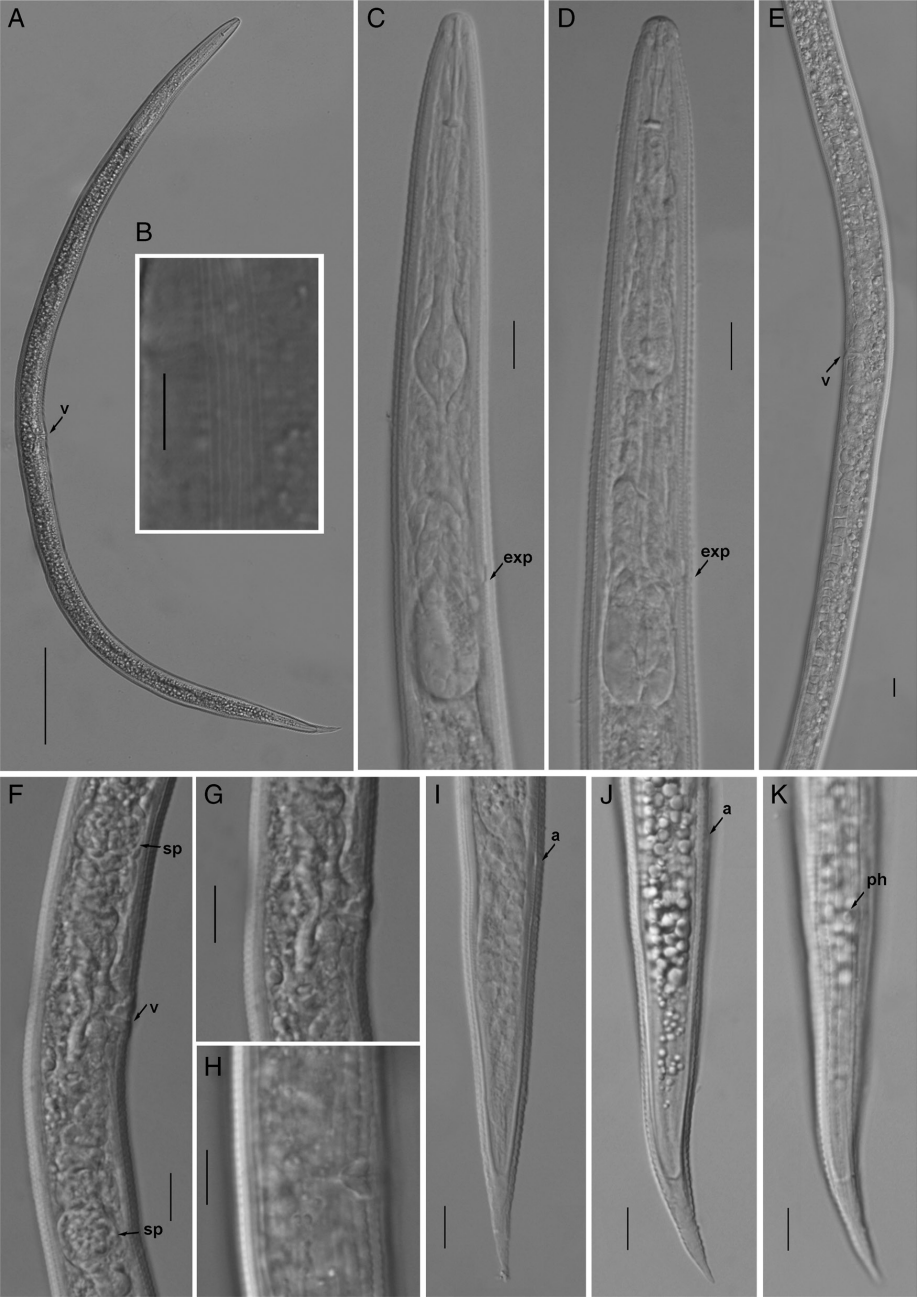
Light photomicrographs of *Geocenamus chengi* n. sp. Female. A: entire body; B: lateral lines; C, D: pharyngeal regions, arrow pointing on the excretory pore (exp): E: gonad; F: vulval region arrows pointing on vulva (v) and spermatheca (sp); G, H: vulval region; I-K: female tails arrows pointing on anus (a) and phasmid (ph) (Scale bars = A = 100 μm; B-K = 10 μm).

**Figure 3:**
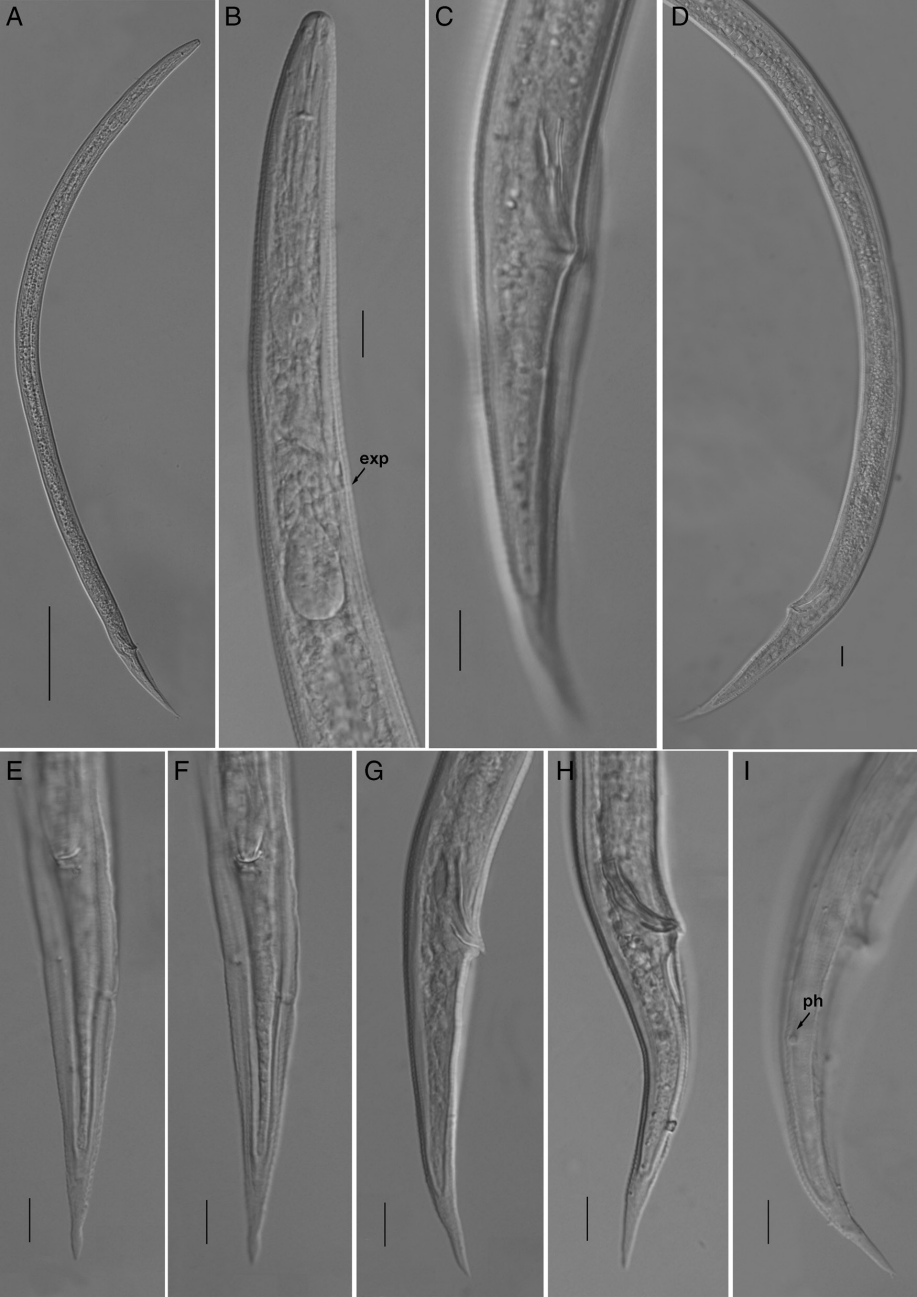
Light photomicrographs of *Geocenamus chengi* n. sp. Male. A: entire body; pharyngeal regions, arrow pointing on the excretory pore (exp); C: tail with bursa; D: gonad; E, F: tail in ventral view; G, I: male tail arrows pointing on phasmid (ph) (Scale bars = A = 100 μm; B-I = 10 μm).

**Figure 4:**
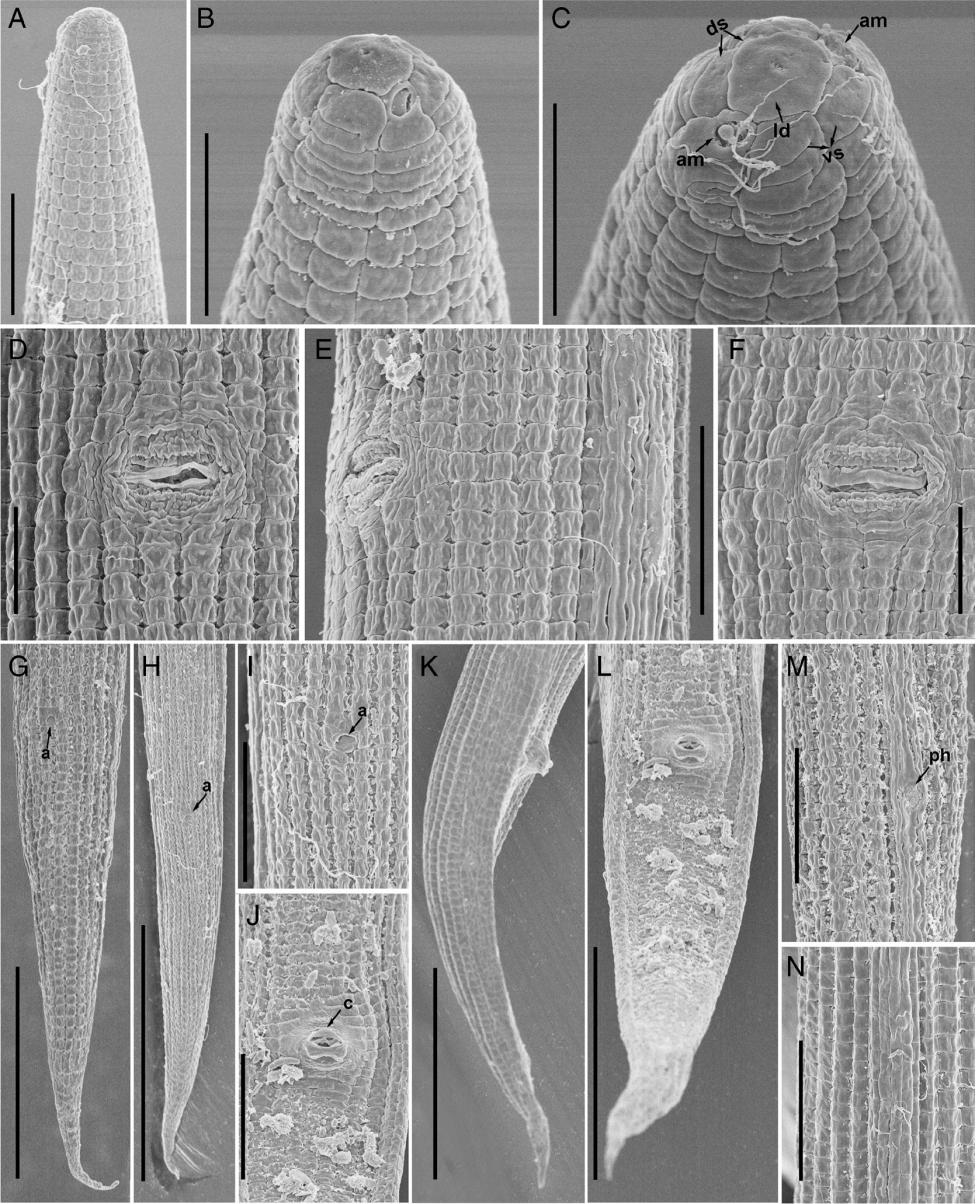
Scanning electron microscopy of *Geocenamus chengi* n. sp. A: anterior region; B-C: en face view; D-F: vulval regions; G, H: female tail regions arrows pointing anus (a); I: anal area at higher resolution, arrow pointing on the position of anus; J: cloacal area at higher resolution, arrow pointing on the position of cloaca (c); K, L: male tail; M: lateral field with phasmid (ph); N: lateral field. Abbreviation: am, apmphid; ds, dorsal sectors; vs, =ventral sectors; ld, labial disc (Scale bars, A, E, I, J, M, N = 10 μm; B, C, D, E, F = 5 μm; G, K = 30 μm; H = 50 μm; L = 20 μm).

**Table 1.** Morphometric data for *Geocenamus chengi* n. sp. All measurements are in μm and in the form of mean ± SD (range).

	Holotype	Paratype	
		Female	Male
*n*		20	10
Body length	884.8	879.0 ± 59.6 (783–997)	822.8 ± 34.2 (772–892)
a	33.3	33.3 ± 2.6 (28–39)	32.9 ± 1.2 (31–34)
b	6.0	6.1 ± 0.3 (5.5–6.6)	6.1 ± 0.2 (5.8–6.6)
c	10.4	10.5 ± 0.6 (9.2–11.7)	9.7 ± 0.6 (8.7–10.3)
c'	4.7	4.6 ± 0.4 (4.2–5.5)	4.8 ± 0.3 (4.5–5.2)
V	52.8	52.5 ± 1.6 (49.5–55.7)	39.4 ± 5.4 (33.1–47.4)
Lip height	7.5	7.6 ± 2.4 (7.0–8.0)	7.6 ± 0.3 (7.0–8.0)
Lip width	3.7	3.8 ± 0.3 (3.4–4.5)	3.7 ± 0.3 (3.2–4.0)
Stylet length	22.3	22.2 ± 0.6 (21–23)	21.5 ± 0.7 (20–23)
DGO distance from stylet knobs	2.0	2.0 ± 0.1 (1.7–2.2)	2.1 ± 0.1 (1.9–2.3)
Excretory pore from anterior end	122.8	120.6 ± 5.2 (109–126)	108.3 ± 5.2 (101–116)
Hemizonid from anterior end	118.4	116.7 ± 5.1 (104–123)	103.0 ± 6.7 (94–113)
Pharynx length	148.4	145 ± 5.7 (130–152)	134 ± 3.6 (127–139)
Maximum body diam.	26.6	26.6 ± 2.5 (22–33)	25.0 ± 1.0 (23–27)
Vulval body diam.	25.4	26.6 ± 2.3 (23–32)	−
Anal/cloacal body diam.	18.3	18.1 ± 1.2 (16–21)	17.7 ± 0.9 (16–19)
Tail length	85.2	83.8 ± 5.4 (76–92)	85.2 ± 5.4 (78–93)
Hyaline tail part length	25.2	22.4 ± 2.2 (19–25)	21.3 ± 1.9 (18–24)
Phasmid position from anus/cloaca	12.4	15.5 ± 4.1 (10–19)	24.6 ± 3.9 (19–29)
Phasmid % of tail	14.6	17.4 ± 4.0 (12–20)	29.5 ± 4.0 (24–35)
Spicule length		−	23.4 ± 1.9 (22–25)
Gubernaculum		−	8.0 ± 0.7 (7–9)

The status of genera in subfamily Merliniinae (Siddiqi, 1971) has been discussed by several nematologists, namely Andrássy (1977), Hooper (1978), Fortuner and Luc (1987), Siddiqi (1986, 2000), and Brzeski (1991). However, based on the recent classification (Geraert, 2011), the subfamily Merliniinae contains *Amplimerlinius* (Siddiqi, 1976), *Geocenamus*, and *Nagelus* (Thorne and Malek, 1968) species. The rest of the genera *Scutylenchus* (Jairajpuri, 1971), *Merlinius* (Siddiqi, 1979), *Hexadorus* (Ivanova and Shagalina, 1983), *Pathotylenchus* (Eroshenko and Volkova, 1987), and *Allentylenchus* (Khan and Saeed, 1988) were considered as junior synonyms of *Geocenamus* (Geraert, 2011).

Currently, the genus *Geocenamus* contains over 70 species distributed across different climatic zones and environments (Geraert, 2011; Nguyen et al., 2019). *G. brevicaudatus* (Peng and Hunt, 1995; Brzeski, 1998) is a Chinese native species, and was reported almost two decades ago from Hebei province. Other than that, *G. myunsugae* (Choi and Geraert, 1993) and *G. tenudens* (Thorne and Malek, 1968) were also reported in the rhizosphere of cultivated plants from Shandong and Liaoning provinces, respectively (Ni and Liu, 2004; Li et al., 2004). Since then, none of the *Geocenamus* have ever been reported from China; considering the scarce occurrence of the *Geocenamus* species in China, the detected species was characterized morphologically (using light and scanning microscopy) and molecularly (with 18 S, 28 S, and ITS genes sequences). The morphometrics and morphological characters of the detected species were compared with the related *Geocenamus* species, and it found that this species possesses unique characters and needs to be considered as a new member of the genus. Therefore, this study describes a new *Geocenamus* species with the following objectives: to provide an integrative morphological and molecular characterization of the new species; to elucidate important morphological details through SEM observations; and to study the phylogenetic relationships of these species with other merlinid and related nematodes.

## Materials and methods

### Nematode extraction and morphological study

Nematodes were extracted from soil and root samples using the modified Cobb sieving and flotation-centrifugation method (Jenkins, 1964). For morphometric studies, nematodes were killed and fixed in hot formalin (4% with 1% glycerol) and processed in glycerin (Seinhorst, 1959). The measurements and light micrographs of nematodes were made with a Nikon Eclipse Ni-U 931845 compound microscope. For the SEM examination, the nematodes were fixed in a mixture of 2.5% paraformaldehyde and 2.5% glutaraldehyde, washed three times in 0.1 M cacodylate buffer, post-fixed in 1% osmium tetroxide, dehydrated in a series of ethanol solutions, and critical point-dried with CO_2_. After mounting on stubs, the samples were coated with gold at 6 to 10 nanometer thickness, and the micrographs were made at 3 to 5 kV operating system (Maria et al., 2018).

### Molecular analyses

DNA was extracted by transferring individual nematodes into an Eppendorf tube containing 16 μL ddH_2_O. Nematodes were crushed using a sterilized pipette tip, and the tubes were centrifuged at 12,000 rpm for 1 min and frozen at −68°C for at least 30 min. Tubes were heated to 85°C for 2 min, and then, 2 μL proteinase K was added in PCR buffer solution. The tubes were incubated at 56°C for 1 to 2 hr and at 95°C for 10 min. After incubation, these tubes were cooled to 4°C and used for conducting PCR analyses (Zheng et al., 2003). Several sets of primers (synthesized by Invitrogen, Shanghai, China) were used in the PCR analyses to amplify the partial 18 S, ITS region and D2-D3 expansion domains of 28 S of rDNA. Primers for amplification of partial 18 S were 18s900-18s1713 (Olson et al., 2017). Primers for amplification of ITS were TW81-AB28 (Joyce et al., 1994). The primers for amplification of D2-D3 of 28 S were D2A and D3B (De Ley et al., 1999). PCR conditions were as described by Ye et al. (2007) and Powers et al. (2010). PCR products were evaluated on 1% agarose gels stained with ethidium bromide. PCR products of sufficiently high quality were sent for sequencing by Invitrogen (Shanghai, China).

### Phylogenetic analysis

The newly obtained sequences were deposited into the GenBank database, and accessions were in the phylogenetic trees. The DNA sequences were compared with those of the other merlinids and related nematodes available at the GenBank sequence database using the BLAST homology search program. Outgroup taxa for the data set were chosen according to previously published data (Handoo et al., 2014; Nguyen et al., 2019). Multiple alignments of the different sequences were made using the Q-INS-i algorithm of MAFFT v. 7.205 (Katoh and Standley, 2013). The best-fit model of DNA evolution was obtained using jModelTest V.2.1.7 (Darriba et al., 2012) with the Akaike information criterion (AIC). The best-fit model, the base frequency, the proportion of invariable sites, and the gamma distribution shape parameters and substitution rates in the AIC were then given to MrBayes for the phylogenetic analyses. Transitional model and gamma-shaped distribution (TIM3ef + G) were used for the 18 S; unlinked general time-reversible model with invariable sites and a gamma-shaped distribution (GTR + I + G) was used for D2-D3 expansion domains of 28 S, and transitional model with invariable sites and a gamma-shaped distribution ( TIM2 + I + G) for ITS. Bayesian analysis was performed to confirm the tree topology for each gene separately using MrBayes 3.1.0 (Huelsenbeck and Ronquist, 2001) with four chains for 2 × 10^6^ generations. The Markov chains were sampled at intervals of 100 generations. Two runs were conducted for each analysis. After discarding burn-in samples of 10% and evaluating convergence, the remaining samples were retained for more in-depth analyses. The topologies were used to generate a 50% majority-rule consensus tree. Posterior probabilities (PP) are given on appropriate clades. Trees from all analyses were edited by FigTree software V.1.4.4 (http://tree.bio.ed.ac.uk/software/figtree/).

## Results and description

### Systematics

*Geocenamus chengi* n. sp. (Figs. 1-4; Table 1).

### Description

#### Female

After fixation, the body is ventrally curved or C shaped. There are six incisures on lateral field, areolation at mid-body and tail region is observed in the majority of individuals. Body annuli are clearly defined and divided into blocks (seen under SEM). Labial region is dome shaped and slightly offset from the rest of the body having four to five annuli. Irregular rounded rectangular labial disc is observed surrounded by the dorsal, ventral sectors, and amphidial apertures. The labial framework is not sclerotized. Stylet is well-developed with rounded knobs. The dorsal gland orifice is located 2.0 μm posterior to stylet knobs. Median bulb is oval with bean-shaped central valve plates; deirid is absent. Isthmus is slender, surrounded by a nerve ring; pharyngeal basal bulb is saccate and abutting intestine. Cardia is indistinct and conoid rounded. Excretory pore is located at the anterior region of basal pharyngeal bulb. Hemizonid is 2-3 annuli long, anterior to excretory pore; vulva is a transverse slit, vulval lips are elongated and ellipsoidal with epiptygma (SEM), and vagina is “v” shaped comprising less than half of the corresponding diameter; spermatheca is rounded filled with rounded sperm cells; ovaries are outstretched with a single row of oocytes. The tail is annulated, elongated, and conical with a terminal hyaline region, which comprises 21 to 33% of the tail length, ending as bluntly pointed tip. Phasmid is small and pore like, located 10 to 19% posterior from anus.

#### Male

Body habitus, cuticle and anterior region of males are similar to females. Gonad is located on the right side of the intestine and outstretched. Spicule is 22 to 25 μm long with truncated head and having an abrupt depression just below the head on the dorsal side, followed by a curve that tapers till the distal end of the spicule, and distal tips are bluntly pointed; gubernaculum is saucer shaped; cloacal lips are not protuberant, and two posterior hypoptygmata can be seen under SEM. Bursa crenate covers the tail until the hyaline tail region. Tail shape is similar to that of female, and terminal hyaline region comprises 20 to 28% of the tail length. Phasmid is small and located 19 to 29% posterior from cloaca.

### Type host and locality

This population was detected in the rhizosphere of *Camelliae sinensis* (L.) Kuntze, 1887 from Longjian Tea Village, Hangzhou City, Zhejiang Province, P. R. China, on December, 2019.

### Type material

Holotype female and 16 female and 8 male paratypes (slide numbers ZJU-31-01-ZJU-31-10) were deposited in the nematode collection of Zhejiang University, Hangzhou, China. Additional 10 slides having plenty of male and female were also stored in the same collection. Also, 4 females and 2 male paratypes (slide numbers T-7378p, T-7379p) were deposited at USDA nematode collection, Beltsville, Maryland, USA.

### Etymology

The species is named after late Professor Hurui Cheng, one of the famous plant nematologists in China, for his extraordinary contribution to the nematode taxonomy from China.

### Diagnosis and relationships

*Geocenamus chengi* n. sp. can be characterized by females having six incisures in the lateral field; labial region is dome shaped and slightly offset from the rest of the body having four to five annuli; deirids are absent; excretory pore is located at the anterior region of the basal pharyngeal bulb. The vulva is a transverse slit, vulval lips are elongated and ellipsoidal with epiptygma (seen under SEM). The tail is annulated, elongated and conical having bluntly pointed tip and a terminal hyaline region that comprises 21 to 33% of the tail length; phasmid is small and pore like, located 10 to 19% posterior from anus. Spicule is 22 to 25 μm long, gubernaculum is saucer shaped; bursa is crenate covering the tail until the hyaline tail region.

Based on the similar tail morphology, *G. chengi* n. sp. is characterized close to *G. circellus* (Anderson and Ebsary, 1982; Brzeski, 1991), *G. joctus* (Thorne, 1949; Brzeski, 1991); *G. loofi* (Siddiqi, 1979; Brzeski, 1991), *G. ordinarius* (Volkova, 1993), *G. processus* (Siddiqi, 1979; Brzeski, 1991), *G. tetyllus* (Anderson and Ebsary, 1982), and *G. tortilis* (Kazachenko, 1980; Brzeski, 1991).

From *G. circellus*, it can be differentiated by longer body of females 879 (783-997) vs 520 to 650 μm, longer stylet of females 22 (21-23) vs 9 to 10 μm, longer tail of females 84 (76-92) vs 76 μm, shape of lip region (dome-shaped and slightly set off vs low, truncated, and continuous), deirids (absent vs present), location of excretory pore of females 120.6 (109-126) vs 99 μm, vulval lips (simple *vs* elevated), phasmid position from anus 15 (10-19) vs 23 to 27 μm, and spicule terminus (bluntly pointed vs indented).

From *G. joctus*, it can be differentiated by longer body of females 879.0 (783-997) vs 520 to 790 μm, longer stylet of females 22.2 (21-23) vs 15.5 to 19.5 μm, longer tail of females 84 (76-92) vs 38 to 77 μm, number of incisures in the lateral field 6 vs 10, shape of lip region (dome-shaped and slightly set off vs hemispherical set off by a constriction), deirids (absent vs present), length of hyaline tail terminus of females 22 (19-25) vs 7.5 to 11.5 μm and phasmid position on the tail of females 17.4 (12-20) vs 33 to 58%.

From *G. loofi*, it can be differentiated by longer body of females 879 (783-997) vs 510 to 620 μm, longer stylet of females 22 (21-23) vs 9 to 10 μm, longer tail of females 84 (76-92) vs 62 μm, shape of lip region (dome-shaped and slightly set off vs low, truncated, and continuous), position of excretory pore (at the anterior region of basal pharyngeal bulb vs in the isthmus region), tail terminus (bluntly pointed tip vs mucronated), and phasmid position (anterior half of the tail vs middle of the tail).

From *G. ordinarius,* it can be differentiated by longer stylet of females 22 (21-23) vs 9 to 10 μm, longer tail of females 84 (76-92) vs 60 to 70 μm, shape of lip region (dome-shaped and slightly set off vs hemispherical set off by a constriction), labial disc protruded (absent vs present), position of excretory pore (at the anterior region of basal pharyngeal bulb vs at the level of isthmus base), and phasmid position (anterior half of the tail vs middle of the tail).

From *G. processus*, it can be differentiated by longer body of females 879 (783-997) vs 500 to 630 μm, longer stylet of females 22 (21-23) vs 15.0 to 17.5 μm, longer tail of females 84 (76-92) vs 50 μm, shape of lip region (dome-shaped and slightly set off vs lip region hemispherical set off by a depression), position of excretory pore (at the anterior region of basal pharyngeal bulb vs at the level of isthmus base), tail terminus (bluntly pointed tip vs mucronated), and spicule terminus (bluntly pointed vs notched).

From *G. tetyllus*, it can be differentiated by stylet length of females 22 (21-23) vs 14 μm, shape of lip region (dome-shaped and slightly set off vs subtruncated, set off by a constriction), deirids (absent vs present), phasmid position from anus 15 (10-19) vs 42 to 46 μm, and spicule terminus (bluntly pointed vs notched).

From *G. tortilis*, it can be differentiated by longer body of females 879 (783-997) vs 480 to 540 μm, stylet length of females 22 (21-23) vs 19.5 μm, longer tail of females 84 (76-92) vs 54 to 58 μm, shape of lip region (dome-shaped and slightly set off vs rounded set off by a depression), position of excretory pore (at the anterior region of basal pharyngeal bulb vs at the level of nerve ring), and spicule length 23 (22-25) vs 27 μm.

### Molecular profiles and phylogenetic status

*Geocenamus chengi* n. sp. was molecularly characterized using partial 18 S, D2-D3 expansion domains of 28 S and ITS sequences. As few species of the genus have been provided with sequence-based information, all the available sequences of subfamily Merliniinae deposited in the GenBank were included in the phylogenetic analysis.

**Figure 5:**
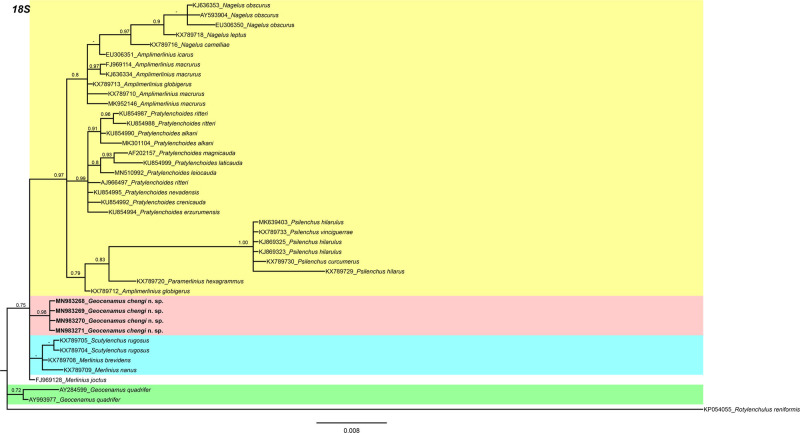
Phylogenetic relationships of *Geocenamus chengi* n. sp. with other merlinids as inferred from Bayesian analysis using the 18 S rRNA gene sequence data set with the TIM3ef + G model. Posterior probability more than 70% is given for appropriate clades. Newly obtained sequences are indicated in bold.

**Figure 6:**
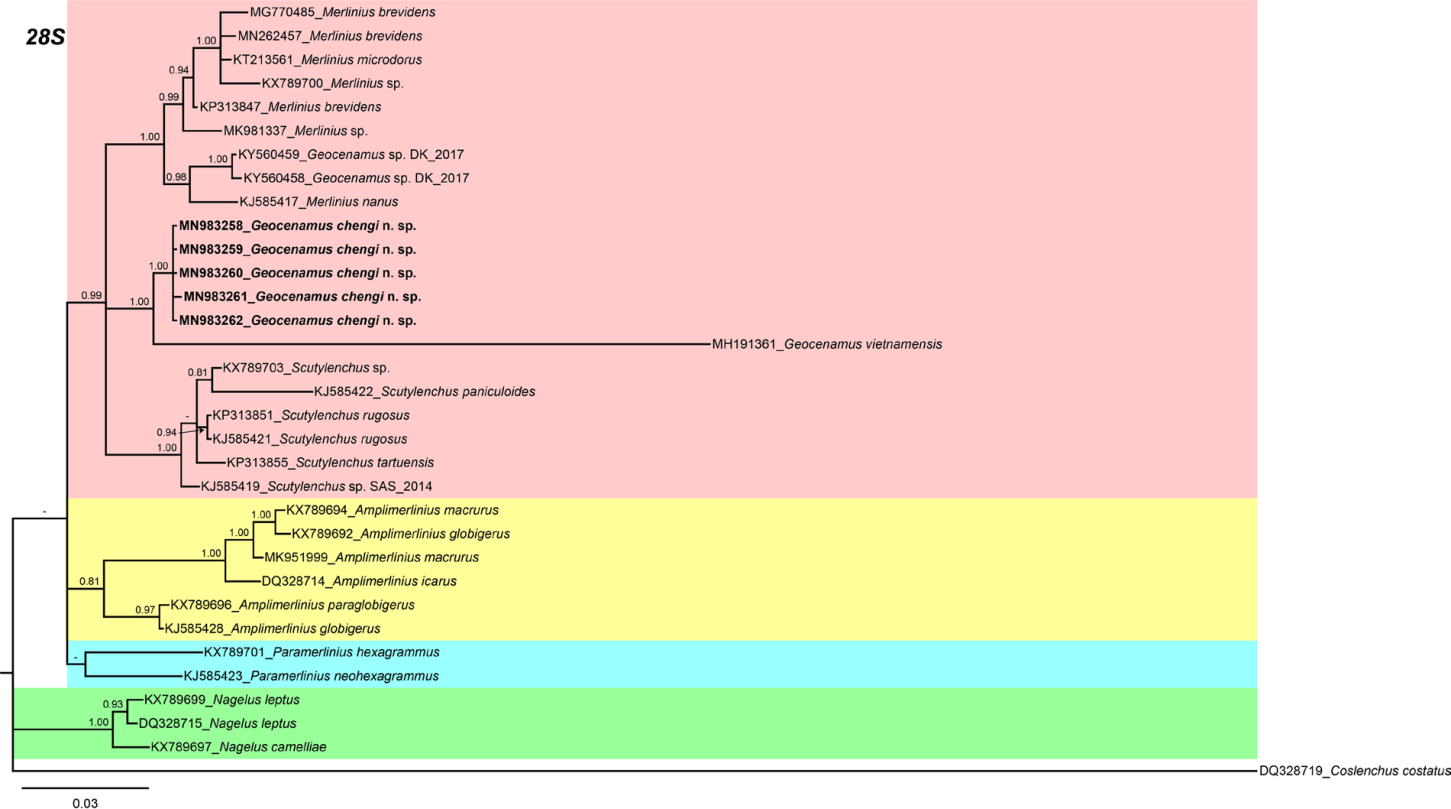
Phylogenetic relationships of *Geocenamus chengi* n. sp. with other merlinids as inferred from Bayesian analysis using the D2-D3 of 28 S rRNA gene sequence data set with the GTR + I + G model. Posterior probability more than 70% is given for appropriate clades. Newly obtained sequences are indicated in bold.

**Figure 7:**
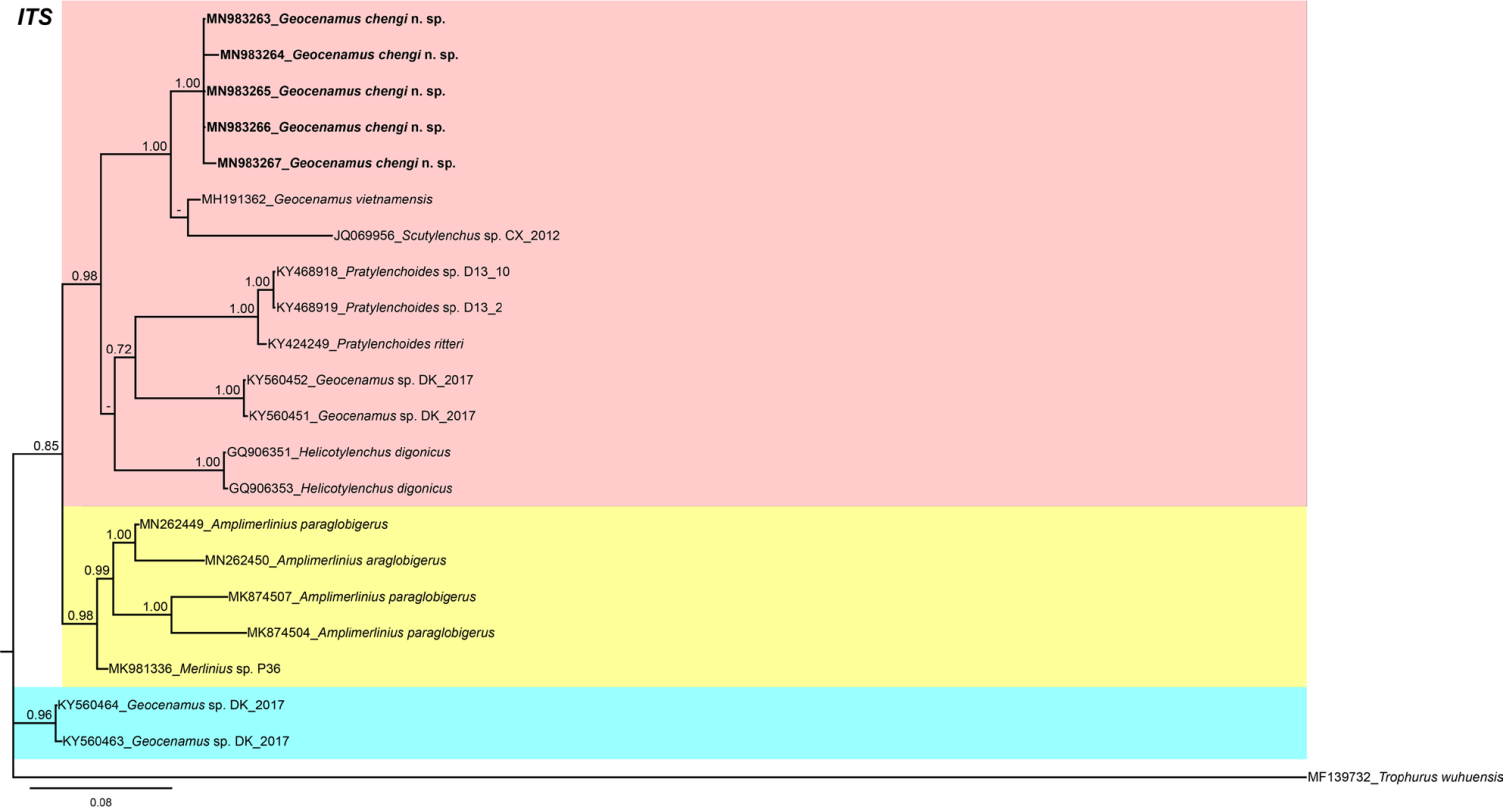
Phylogenetic relationships of *Geocenamus chengi* n. sp. with other merlinids as inferred from Bayesian analysis using the ITS rRNA gene sequence data set with the TIM2 + I + G model. Posterior probability more than 70% is given for appropriate clades. Newly obtained sequences are indicated in bold.

In the 18 S gene analysis (Fig. 5), the *G. chengi* n. sp. (MN983268-MN983271) forms a separate clade next to *Scutylenchus rugosus* (KX789704-KX789705), *Merlinius brevidens* (KX789708), and *M. nanus* (KX789709). In the 28 S gene tree (Fig. 6), *G. chengi* n. sp. (MN983258-MN983262) clustered with *G. vietnamensis* (MH191361) in a well-supported subclade (PP = 1.00), whereas the other members of subfamily Merliniinae arranged in separate clades. In the ITS gene tree (Fig. 7), *G. chengi* n. sp. (MN983263-MN983267) clustered with *G. vietnamensis* (MH191362) and an unidentified *Scutylenchus* (JQ069956) species from China.

In all the phylogenetic analyses, *G. chengi* n. sp. forms a separate clade and grouped with *G. vietnamensis*. The sequence identities of the new species with *G. vietnamensis* are 95% (25 nucleotide, 2 indels differences) for 28 S, and 94% (39 nucleotide, 14 indels differences) for ITS. Morphologically, *G. chengi* n. sp. can be differentiated from *G. vietnamensis* by the posterior position of the excretory pore (vs at nerve ring level), short tail (vs 85-125 μm), and shorter stylet (vs 24-28 μm).

## Discussion

The classification presented by Siddiqi (2000) and Andrássy (2007) distinguished *Geocenamus*, *Nagelus*, *Merlinius*, *Scutylenchus*, and *Amplimerlinius* within the subfamily Merliniinae. Fortuner and Luc (1987) and Maggenti et al. (1988) placed the genus *Geocenamus* within the subfamily Belonolaiminae (Whitehead, 1959), whereas Bongers (1988) arranged *Merlinius*, *Amplimerlinius*, *Scutylenchus*, *Geocenamus*, and *Nagelus* in the family Dolichodoridae (Chitwood, 1950). In a recent classification by Geraert (2011), he accepted the synonymization of *Merlinius* and *Scutylenchus* with *Geocenamus* and retained only the genera *Amplimerlinius*, *Geocenamus*, and *Nagelus* in subfamily Merliniinae. On contrary to this, Sturhan (2012) agreed with Brzeski (1991) in synonymizing *Scutylenchus* with the *Geocenamus*, but he considered *Merlinius* as a valid genus.

In our phylogenetic analysis, *Geocenamus*, *Scutylenchus*, and *Merilinius* appeared as distinct genera supporting Siddiqi’s (2000) classification, similar results were obtained in several other studies (Carta et al., 2010; Ghaderi et al., 2014; Nguyen et al., 2019). It is also noted that the available *Geocenamus* species do not group with the new species and *G. vietnamensis*, and in this context, we agreed with Carta et al. (2010) who stated that greater genetic distance between species or *Geocenamus* populations possibly due to the presence of cryptic species, different haplotypes or misidentification. Other than that, the majority of Merliniinae genera and species have not yet been sequenced, and we expect, with the inclusion of additional/new sequences of Merliniinae the phylogenetic studies could provide better insights than now.

The present study does not address any taxonomic revisions or higher classification; however, it describes a new *Geocenamus* species isolated from the tea plantations of Hangzhou City. This is the first *Geocenamus* species associated with tea in China, no obvious aboveground or root symptoms were detected on the plants. Other than *G. chengi* n. sp., the most numerous plant-parasitic nematodes were the ring nematode, i.e. *Hemicricoemoides chitwoodi*. No other plant-parasitic taxa were detected, except some predaceous, microbivores, and fungivore nematodes. Currently, *Geocenamus* contains over 70 species and 38 of them were described/reported from Asia (Geraert, 2011; Nguyen et al., 2019). The high level of *Geocenamus* diversity may indicate that a relatively rapid speciation rate of this nematode group occurred in this region; however, additional studies are required to shed light on the evolution, phylogeny, and ecological aspects of these nematodes.

## References

[ref001] Anderson R. V. and Ebsary B. A. 1982 Canadian species of *Merlinius Siddiqi*, 1970 and a diagnosis and description for *Mulveyotus hyalacus* n. gen., n. sp. (Nematoda: Tylenchorhynchidae). Canadian Journal of Zoology 60:521–529.

[ref002] Andrássy I. 1977 *Tylenchus davainei*. C.I.H. Descriptions of Plant-Parasitic Nematodes, Set 7, No. 97 Commonwealth Agricultural Bureaux, Farnham Royal.

[ref003] Andrássy I. 2007 Free-living nematodes of Hungary (*Nematoda errantia*). Vol. II. Hungarian Natural History Museum. Budapest, Hungary 496 pp.

[ref004] Brzeski M. W. 1991 Taxonomy of *Geocenamus* Thorne & Malek, 1968 (Nematoda: Belonolaimidae). Nematologica 37:125–173.

[ref005] Bongers T. 1988 De Nematoden van Nederland Koninklijke Natuurhistorische Vereniging, Utrecht, 408 pp.

[ref006] Carta L. K., Skantar A. M. and Handoo Z. A. 2010 Molecular rDNA phylogeny of Telotylenchidae Siddiqi, 1960 and evaluation of tail termini. Journal of Nematology 42:359–369. 22736870 PMC3380519

[ref050] Chitwood B. G. 1950 “General structure of nematodes”, in Chitwood B. G. and Chitwood M. B. (Eds), An Introduction to Nematology. Section 1, Anatomy. Monumental Printing Co., Baltimore, pp. 7–187.

[ref007] Choi Y. E. and Geraert E. 1993 Nematodes associated with forest trees in Korea. II: three new and one described species of *Geocenamus* with a note on the en face view in the genus. Nematologica 39:431–449.

[ref008] Darriba D., Taboada G. L., Doallo R. and Posada D. 2012 jModelTest 2: more models, new heuristics and parallel computing. Nature methods 9:772. 10.1038/nmeth.2109 PMC4594756 22847109

[ref010] De Ley P., Félix M. A., Frisse L. M., Nadler S. A., Sternberg P. W. and Thomas W. K. 1999), Molecular and morphological characterization of two reproductively isolated species with mirror-image anatomy (Nematoda: Cephalobidae). Nematology 1:591–612.

[ref011] Eroshenko A. S. and Volkova T. V. 1987 Nematodes *Geocenamus* *patternus* n.sp. and *Patholotylenchus nurserus* n.gen.et. n.sp. from rhizosphere of Coniferales of the Far east of the USSR. Parazitologijia 21:595–598.

[ref012] Fortuner R. and Luc M. 1987 A reappraisal of Tylenchina (Nemata): 6. The family Belonolaimidae Whitehead, 1960. Revue de Nématologie 10:183–202.

[ref013] Geraert E. 2011 The Dolichodoridae of the World: Identification of the Family Dolichodoridae (Nematoda: Tylenchida) Academia Press, Gent, 520 pp.

[ref014] Ghaderi R., Karegar A., Niknam G. and Subbotin S. A. 2014 Phylogenetic relationships of Telotylenchidae Siddiqi, 1960 and Merliniidae Siddiqi, 1971 (Nematoda: Tylenchida) from Iran, as inferred from the analysis of the D2–D3 expansion fragments of 28S rRNA gene sequences. Nematology 16:863–877.

[ref016] Handoo Z. A., Palomares-Rius J., Cantalapiedra-Navarrete E., Liébanas C., Subbotin G. S. A. and Castillo P. 2014 Integrative taxonomy of the stunt nematodes of the genera *Bitylenchus* and *Tylenchorhynchus* (Nematoda, Telotylenchidae) with description of two new species and molecular phylogeny. Zoological Journal of the Linnean Society 172:231–264.

[ref017] Hooper D. J. 1978 “Structure and classification of nematodes”, in Southey J. F. (Ed.), Plant Nematology Her Majesty’s Stationery Office, London, pp. 3–45.

[ref018] Huelsenbeck J. P. and Ronquist F. 2001 MrBAYES: Bayesian inference of phylogenetic trees. Bioinformatics 17:754–755. 11524383 10.1093/bioinformatics/17.8.754

[ref019] Ivanova T. S. and Shagalina L. M. 1983 A new genus and species of the nematode *Hexadorus deserticola* n.g., n.sp. (Tylenchida, Belonolaiminae) in deserts of Central Asia. (In Russian), Parazitologiya 17:318–321.

[ref020] Jairajpuri M. S. 1971 On Scutylenchus mamillatus (Tobar-Jiménez, 1966) n.comb. 40th annual session. Proceedings of the National Academy of Sciences India, 18 pp.

[ref021] Jenkins W. R. 1964 A rapid centrifugal-flotation technique for separating nematodes from soil. Plant Disease Reporter 48:692.

[ref022] Joyce S., Reid A., Driver F. and Curran J. 1994 “Application of polymerase chain reaction (PCR) methods to identification of entomopathogenic nematodes”, in Burnell A. M., Ehlers R. U. and Masson J. P. (Eds), COST 812 Biotechnology: Genetics of Entomopathogenic Nematode-bacterium Complexes. Proceedings of Symposium & Workshop, St. Patrick’s College, Maynooth, Co European Commission, DG XII, Kildare, pp. 178–187.

[ref023] Katoh K. and Standley D. M. 2013 MAFFT multiple sequence alignment software version 7: improvements in performance and usability. Molecular Biology and Evolution 30:772–780. 23329690 10.1093/molbev/mst010 PMC3603318

[ref024] Kazachenko I. P. 1980 New species of nematodes (Tylenchida) and a description of the male *Yeratocephalus sigillarius* (Rhabditida) from forests in the Far East. Zoologichesky Zhurnal 59:810–817.

[ref025] Khan A. and Saeed 1988 Studies on some members of the family Tylenchorhynchidae, wih comments on *Tylenchorhynchus obscurisulatus* Andrassy, 1959, *T. paranudus* Phukan & Sanwal, 1982 and *Merlinius varians* (Thorne & Malek, 1968), Siddiqi, 1970. Bangladesh Journal of Scientific and Industrial Research 23:74–79.

[ref027] Li J. H., Duan Y. X. and Chen L. J. 2004 Description of one New Recorded Species of Belonolaimidae in China. (In Chinese), Journal of Shenyang Agricultural University 35:101–104.

[ref028] Maggenti A. R., Luc M., Raski D. J., Fortuner R. and Geraert E. 1988 A reappraisal of Tylenchina (Nemata): 11. List of generic and supra-generic taxa, with their junior synonyms. Revue de Nematologie 11:177–188.

[ref029] Maria M., Cai R., Castillo P. and Zheng J. 2018 Morphological and molecular characterization of *Hemicriconemoides paracamelliae* sp. n. (Nematoda: Criconematidae) and two known species of *Hemicriconemoides* from China. Nematology 20:403–422.

[ref030] Ni X. M. and Liu W. Z. 2004 The Description of new recorded species of nematode, *Scutylenchus myungsugae*, in China. (In Chinese), Journal of Laiyang Agricultural College 21:88–90.

[ref031] Nguyen H. T., Linh-Le T. M., Nguyen T. H., Liebanas G., Duong-Nguyen T. A. and Trinh Q. P. 2019 Description of *Geocenamus vietnamensis* sp. n. (Nematoda: Merliniidae) from Vietnam. Journal of Nematology 51:1–12, available at: 10.21307/jofnem-2019-025 PMC6929646 31132005

[ref032] Olson M., Harris T., Higgins R., Mullin P., Powers K., Olson S. and Powers T. O. 2017 Species delimitation and description of *Mesocriconema nebraskense* n. sp (Nematoda: Criconematidae), a morphologically cryptic, parthenogenetic species from North American Grasslands. Journal of Nematology 49:42–66. 28512377 10.21307/jofnem-2017-045 PMC5411254

[ref033] Peng D. L. and Hunt D. J. 1995 *Scutylenchus brevicadatus* sp. nov. from China and *Scutellonema paludosum* sp. nov. (Nematoda: Tylenchida) from the Falkland Islands. Afro-Asian Journal of Nematology 5:55–60.

[ref051] Powers T. O., Harris T., Higgins R., Sutton L., and Powers K. S. 2010 Morphological and molecular characterization of *Discocriconemella inarata*, an endemic nematode from North American native tallgrass prairies. Journal of Nematology 42:35–45. 22736835 PMC3380506

[ref035] Seinhorst J. W. 1959 A rapid method for the transfer of nematodes from ﬁxative to anhydrous glycerin. Nematologica 4:67–69.

[ref036] Siddiqi M. R. 1971 Structure of the esophagus in the classification of the superfamily Tylenchoidea (Nematoda). Indian Journal of Nematology 1:25–43.

[ref037] Siddiqi M. R. 1976 New plant nematode genera *Plesiodorus* (Dolichodorinae), *Meiodorus* (Meiodorinae subfam. n.), *Amplimerlinius* (Merliniinae) and *Gracilancea* (Tylodoridae grad. n.). Nematologica 22:390–416.

[ref038] Siddiqi M. R. 1979 Taxonomy of the plant nematode subfamily Merliniinae Siddiqi, 1970, with descriptions of *Merlinius processus* n. sp., *M. loofi*, n. sp., and *Amplimerlinius globigerus* n. sp. from Europe. Systematic Parasitology 1:43–59.

[ref039] Siddiqi M. R. 1986 Tylenchida Parasites of Plants and Insects 1st ed., CABI Publishing, Wallingford.

[ref040] Siddiqi M. R. 2000 Tylenchida Parasites of Plants and Insects 2nd ed., CABI Publishing, Oxon: 864.

[ref042] Sturhan D. 2012 Contribution to a revision of the family Merliniidae Ryss, 1998, with proposal of *Pratylenchoidinae* subfam. n., *Paramerlinius* gen. n., *Macrotylenchus* gen. n. and description of *M. hylophilus* sp. n. (Tylenchida). Journal of Nematode Morphology and Systematics 15:127–147.

[ref043] Thorne G. 1949 On the classification of the *Tylenchida*, new order (Nematoda, Phasmidia). Proceedings of Helminithological Society 16:37–73.

[ref044] Thorne G. and Malek R. B. 1968 Nematodes of the northern great plains. part I. Tylenchida (Nemata: Secernentea). Technical Bulletin of South Dakota Agricultural Experiment Station 111 pp.

[ref045] Volkova T. V. 1993 Root parasitic nematodes of family Tylenchorhnchidae from the world fauna. Book 1, Part I and II: Book 2. Dal’nevostochnoe Otdelenie, Akadeiya Nauk SSSR, Vladivostok, 167+274+171 pp.

[ref046] Whitehead A. G. 1959 *Trichotylenchus falciformis* n. g, n. sp., (Belonolaiminae n. subfam.: Tylenchida Thorne, 1949) an associate of grassroots (*Hyparrhenia* sp.) in Southern Tanganyika. Nematologica 4:279–285.

[ref047] Yao M. Z. and Chen L. 2012 Tea germplasm and breeding in China. Global Tea Breeding 13–68.

[ref048] Ye W., Giblin-Davis R. M., Braasch H., Morris K. and Thomas W. K. 2007 Phylogenetic relationships among *Bursaphelenchus* species (Nematoda: Parasitaphelenchidae) inferred from nuclear ribosomal and mitochondrial DNA sequence data. Molecular Phylogenetics and Evolution 43:1185–1197. 17433722 10.1016/j.ympev.2007.02.006

[ref049] Zheng J., Subbotin S. A., He S., Gu J. and Moens M. 2003 Molecular characterization of some Asian isolates of *Bursaphelenchus xylophilus* and *B. mucronatus* using PCR-RFLPs and sequences of ribosomal DNA. Russian Journal of Nematology 11:17–22.

